# Supplementary material for: Direct observation of kink evolution due to Hund’s coupling on approach to metal-insulator transition in NiS2−xSex
Source: Nat Commun. 2021 Feb 23;12:1208. doi: 10.1038/s41467-021-21460-5 (PMC7902648; doi:10.1038/s41467-021-21460-5)
Supplement: Supplementary file 1 — Supplementary Information [file 41467_2021_21460_MOESM1_ESM.pdf]

Supplementary Information: Direct observation of kink evolution due to Hund's coupling on approach to metal-insulator transition in $NiS_{2\text{-}x}Se_x$

Bo Gyu Jang,[1,2] Garam Han,[3,4] Ina Park,[1] Dongwook Kim,[1] Yoon Young Koh,[5] Yeong Kwan Kim,[6] Wonshik Kyung,[3,4] Hyeong-Do Kim,[3,4] Cheng-Maw Cheng,[7] Ku-Ding Tsuei,[7] Kyung Dong Lee,[8] Namjung Hur,[8] Ji Hoon Shim,[1,9,10,] Changyoung Kim,[3,4] and Gabriel Kotliar[11]

[1]Department of Chemistry, Pohang University of Science and Technology, Pohang 37673, Korea

[2]Center for High Pressure Science and Technology Advanced Research, Shanghai 201203, China

[3]Center for Correlated Electron Systems, Institute for Basic Science (IBS), Seoul 08826, Korea

[4]Department of Physics and Astronomy, Seoul National University, Seoul 08826, Korea

[5]Max Plank POSTECH Center for Complex Phase Materials, Pohang University of Science and Technology, Pohang 37673, Korea

[6]Department of Physics, KAIST, Daejeon, Korea

[7]National Synchrotron Radiation Research Center, Hsinchu 30076, Taiwan

[8]Department of Physics, Inha University, Incheon 22212, Republic of Korea

[9]Department of Physics, Pohang University of Science and Technology, Pohang 37673, Korea

[10]Division of Advanced Materials Science, Pohang University of Science and Technology, Pohang 37673, Korea

[11]Department of Physics and Astronomy, Rutgers University, New Jersey 08854, USA

Supplementary Note 1: Sample characterization

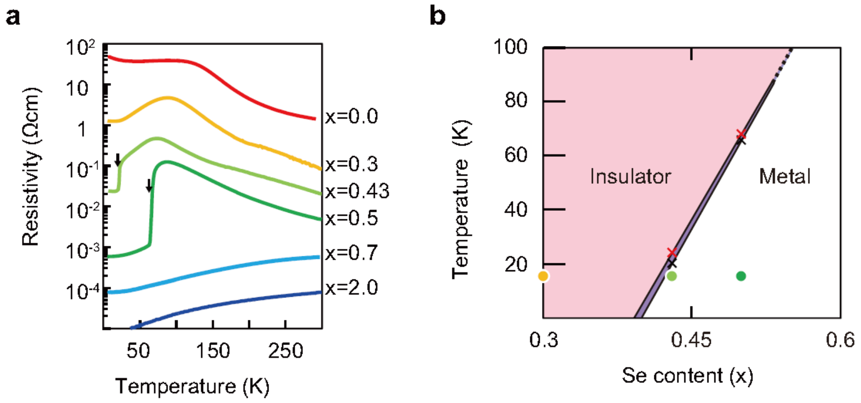

Supplementary Figure 1. Temperature dependent resistivity and phase diagram near the MIT. **a** Temperature dependent resistivity of $NiS_{2-x}Se_x$. Arrows indicate the metal-insulator transition (MIT). **b** A magnified view of the phase diagram near the MIT. It shows how close to the transition measurements were made.

Single crystals of $NiS_{2-x}Se_x$ were grown by chemical vapor transport method using chlorine gas as the transport agent [1, 2]. All single crystals show shiny (100) and (111) facets. Supplementary Table 1 lists Se compositions measured by using energy dispersive X-ray spectroscopy (EDX) as well as corresponding nominal doping values. The values are averages of 3 ~ 5 samples from the same batch. The variation in the measured Se content was smaller than 0.05, which means that all the samples from a batch contain nearly the same Se content. Since the measured doping level was very close to the nominal doping, we use the nominal doping in the main text.

Supplementary Fig.1a shows temperature dependent resistivity curves for x = 0.0, 0.3, 0.43, 0.5, 0.7, and 2.0. The curves for x = 0.0 and 0.3 show an insulating behavior, in contrast to the metallic behaviors seen in the curves for x >= 0.7. The broad hump between 100 K and 150 K shown in the insulating samples is due to the short-range antiferromagnetic order [2]. In the intermediate range between the insulator and metal (x = 0.43 and x = 0.5), the resistivity increases 10 ~ 100 times across the metal-insulator transition (MIT) (see arrows in Supplementary Fig.1a). These results are consistent with published data [2].

The MIT shows a hysteresis behavior and is thus a first order transition. Supplementary Fig.1b is the magnified view of the phase diagram near the MIT obtained based on the resistivity data. The red and black crosses indicate the transition temperatures (T_{MI}) measured while increasing and decreasing the temperature, respectively. The lower bound for x_{MI} is 0.41 at 16 K at which the angle resolved photoemission spectroscopy (ARPES) was taken. This demonstrates that the ARPES data for x = 0.43 shown in Fig. 2d and 2h of the main text represent the Fermi surface and band structure very close to the MIT.

Nominal	0.3	0.43	0.5	0.7	1	1.2
Measured	0.312	0.430	0.516	0.679	1.028	1.256
Std Dev	0.011	0.008	0.019	0.025	0.027	0.047

Supplementary Table 1. Comparison between nominal and measured Se contents. Se content was measured by using energy dispersive X-ray spectroscopy.

Supplementary Note 2: Surface bands of $NiSe_2$

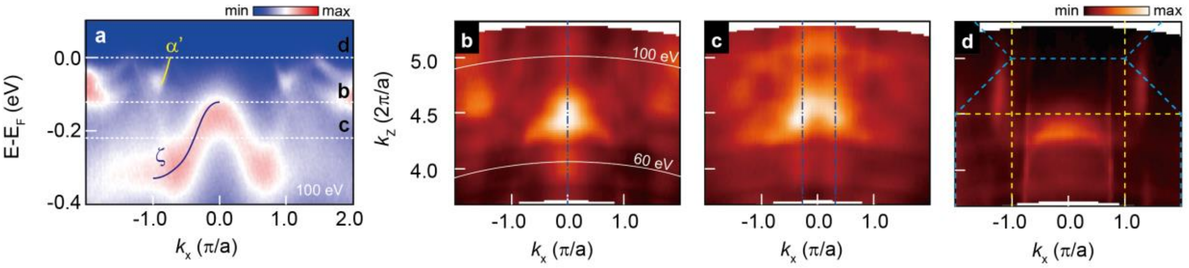

Supplementary Figure 2. Band character of $NiSe_2$ a ARPES data of $NiSe_2$ along the Γ–X direction by using 100-eV photon. **b** and **c**, the constant-energy maps at binding energies indicated by the lines in panel **a**. The dash-dot lines show that the η band is k_z independent. **d** Fermi surface map in the k_x-k_z plane. Yellow dashed-lines indicate the Brillouin zone of the simple cubic unitcell (the same as the ones in Figs. 1a and 1b) while the blue dashed-lines corresponds to the Ni^{2+} fcc sub-unitcell, following the argument in ref. [5].

The noticeable difference between ARPES data and DFT calculation of $NiSe_2$ is a convex band at the Γ point, as indicated by ζ in Fig. 1c and Supplementary Fig. 2a, coming from surface states. To examine the surface-origin of the ζ band, photon energy dependent ARPES is used. Since surface states are localized along the z-axis, surface-state bands should be independent of k_z (i.e, photon energy). Supplementary Fig. 2 shows k_z-dependent ARPES data of $NiSe_2$. Supplementary Fig. 2b and **c** show k_x-k_z constant-energy surface maps at the top (b) and the middle (c) of ζ band, respectively, as indicated by the dotted lines in Supplementary Fig. 2. The data show that the ζ band (guided by blue dash-dotted lines) is independent of the k_z, which suggests that the ζ band has a surface-state origin.

It should be noted that usual surface-states exist usually in the bulk band gap with a narrow peak width because of its long life time. The ζ band does not does not satisfy such conditions. A possible scenario is that the ζ band originates from a surface dead layer [7]. In a Mott system, a surface dead layer can exist where electrons could be more localized due to the low dimensionality. This scenario may be plausible with the observation that the ζ band is present over the entire doping level, even in insulating compounds, as shown in Figs. 2b-2f.

There are other surface-state bands: the surface resonant band α' and electron pockets in the second Brillouin zone. As seen in Supplementary Fig. 2d, the α pocket centered at k_z = 4.0 is clear visible while the band loses its intensity at the k_z=5.0 due to the Ni^{2+} face-centered cubic (fcc) sub-unitcell (the sub-unitcell is indicated by the blue dashed-lines). The α' band is located in the same k region as the α band at k_z = 4.0, thus α' is a surface-resonant band. The electron pockets in the second Brillouin zone also has no k_z dependence. Disappearance of the metallic surface states could be due to breaking the Se-Se chain by inhomogeneity as they disappear when Se is substituted by S as shown in Supplementary Fig. 3a. [8].

Supplementary Note 3: Bulk character of the α band for *x*=0.5

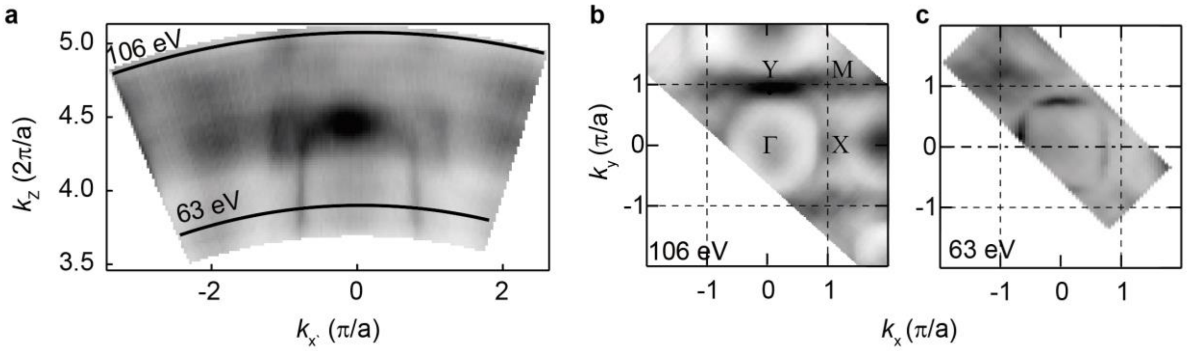

Supplementary Figure 3. Photon-energy-dependent ARPES data along the Γ-X direction for *x*=0.5. a Fermi surface map for *x*=0.5 in the k_x-k_z plane. **b** and **c** Fermi surface maps in the k_x-k_y plane with 106-eV and 63-eV photons, respectively.

There has been a controversy over whether $NiS_{2-x}Se_x$ has metallic surface bands, making surface sensitive low photon-energy ARPES inappropriate to study the MIT. Matsuura *et al.* claimed that the sudden intensity change at E_F across the transition is an evidence for the bulk origin of the quasiparticle peak (QP) [3,4]. On the other hand, a possibility for metallic surface states was proposed based on the fact that a finite spectral weight at E_F even in the insulating phase was observed in the more bulk sensitive SX-ARPES data [5]. However, the QP was more of a shoulder feature of an incoherent spectrum between E_F and -0.4 eV and was not peaky enough to clearly judge the behavior. Therefore, the issue needs to be clearly addressed. The QPs in our spectra are well resolved and can provide an unambiguous answer.

The photon energy and temperature dependent ARPES data for x = 0.5 show that α band contains mostly the bulk character, especially when the system is close to the MIT, and that the band contributes to the MIT. As shown in Supplementary Fig. 3, photon energy dependent ARPES taken with deep ultra-violet light is consistent with the SX-ARPES data (See Fig.1(c) of Ref. [5]). The α band has a rectangular FS in a cubic unit-cell while the overall periodicity follows fcc symmetry due to the Ni^{2+} sub-lattice. The suppression of the intensity above k_z = 4.5 which is due to the fcc sub-lattice is a crucial evidence for bulk-dominant character of the α band.

Supplementary Note 4: Temperature dependent QP behavior

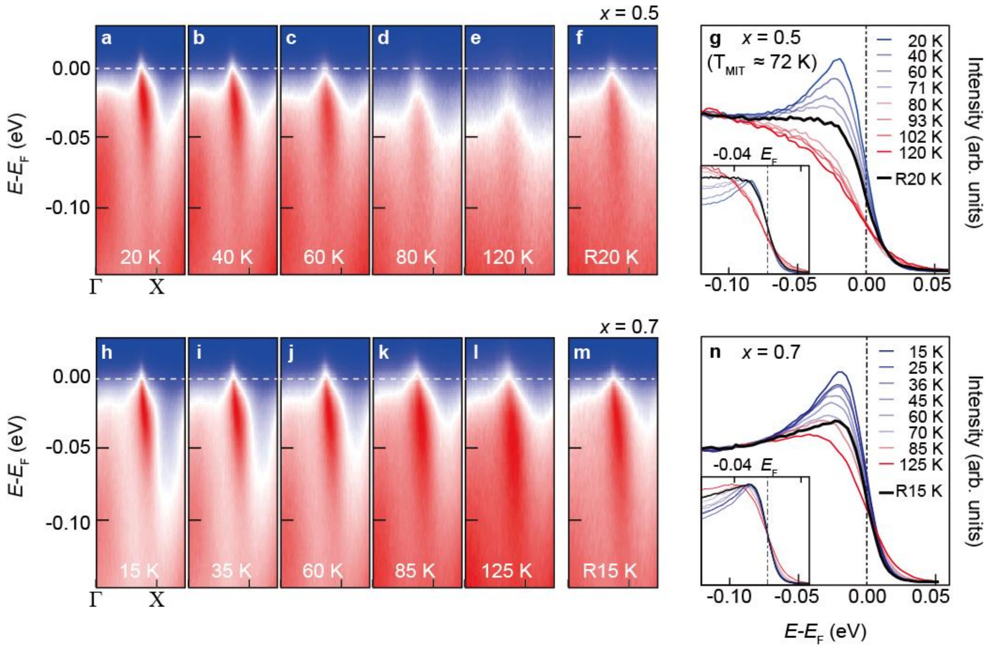

Supplementary Figure 4. Temperature dependent QP behavior. a-e α-band for x = 0.5 taken at various temperatures from 20 to 120 K, **f** after cooling down to 20 K again. **g** Temperature dependent EDCs normalized to background for x = 0.5. The Inset shows the same EDCs but normalized to the peak maximum. R20 K is the data taken after the sample was cooled back down to 20 K. **h-n** The same sequence as in **a-h** but for x = 0.7.

Supplementary Figs. 4a-4f show temperature dependent ARPES spectra from x = 0.5 sample which has a transition temperature (T_{MIT}) of 72 K. As clearly seen in the figures, the α band crosses E_F in the metallic phase below the T_{MIT}. The spectral weight at E_F is considerably reduced once the T increases above the T_{MIT}. Temperature dependent EDCs are displayed in Supplementary Fig. 4g to show the suppression of the QP across the MIT. The QP gradually becomes smaller (due to the reduced hole life time and some surface contamination) as the temperature increases up to T_{MIT} but it abruptly loses its intensity as well as the peaky structure above T_{MIT}. The spectrum does not show further changes even when the temperature is further raised. To check if the disappearance of the QP is due to surface contamination, we lowered the temperature to 20 K again and found that the QP regains the weight as seen in Supplementary Fig 4f and 4g (the black thick line). To demonstrate that such behavior does not occur for a normal metal, data from a metallic sample with x = 0.7 are shown in Supplementary Figs. 4h-4m. The x = 0.7 data do not show such behavior and confirm that the QP behavior is clearly related to the MIT. To our best knowledge, this marks the first clear experimental observation of QP that is directly tied to the Mott transition. In previous studies, the detailed QP near the Fermi level was not clearly discernible as it was buried under incoherent regime above the kink energy scale [3, 4, 5].

Supplementary Note 5: Determination of the quasiparticle dispersion, Fermi velocity, and effective mass from ARPES data

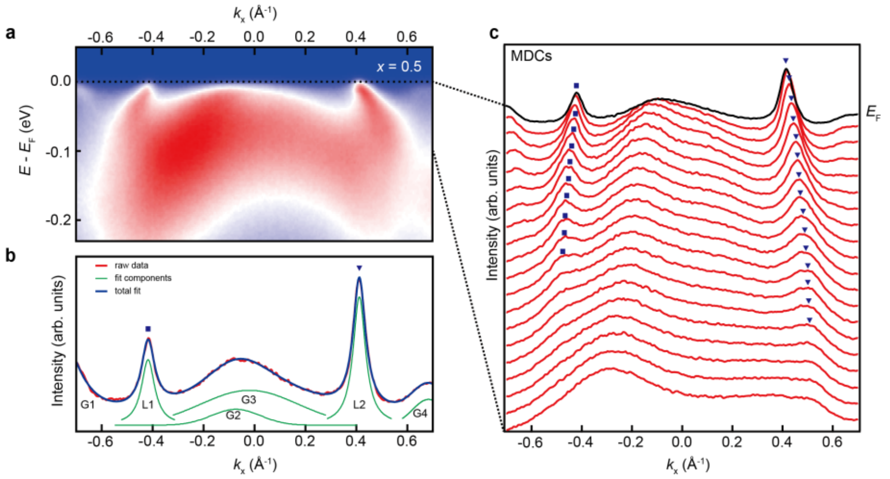

Supplementary Figure 5. Obtaining QP momentum by fitting MDCs. a ARPES data of $NiS_{1.5}Se_{0.5}$ (x=0.5) **b** Momentum distribution curve (MDC) at the Fermi level E_F and the fitting function. **c** Momentum distribution curves (MDCs) from Fermi level to -0.1 eV for x=0.5**.** Squares and inverted triangles indicate the obtained quasiparticle momentum by fitting as shown in panel **b**.

To obtain the quasiparticle dispersion, we used the usual momentum distribution curve (MDC) fitting method [9]. Supplementary Fig. 5 shows the process to determine the quasiparticle dispersion from ARPES data for x = 0.5. A single MDC extracted from ARPES data along the Γ–X direction is well characterized by two Lorentzian peaks (L1 and L2) and four Gaussian peaks (G1, G2, G3 and G4), as shown in Supplementary Fig. 5a and 5b. The fitting should be reasonable since the quasiparticle should have a Lorentzian distribution while backgrounds or insulating bands have Gaussian distributions. The right Lorentzian peak position (inverted triangles) obtained from each MDC is overlaid in Fig. 2d of the main text. The sequence is used in the same way for the other compounds. 'Multipeak fitting 2' of Igor 6.0 from Wave Metrics is used for fitting.

Fermi velocity can be obtained from the slope of QP dispersion at the Fermi level. In order to remove the resolution effect [9] of ARPES, data from Fermi level to ARPES resolution of 15 meV were excluded. v_{F_DFT} was obtained from DFT results for x = 0, 0.5, 1, 1.5 and 2. v_{F_DFT}'s for x = 0.43, 0.7, 1.2 were estimated from linear interpolation.

In Supplementary Fig. 6, we also show the ARPES data and the QP dispersion for x = 1.2 and 1.0. The QP dispersions are shown in Fig. 2h in the main text.

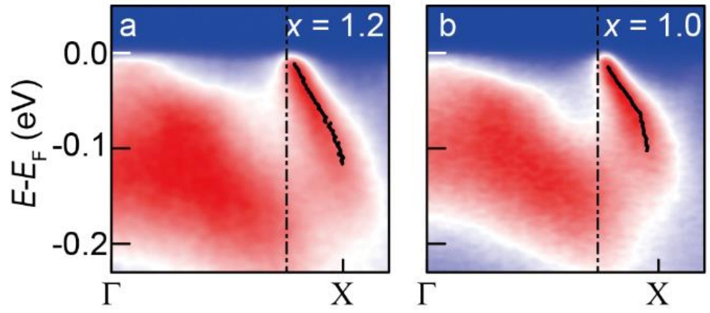

Supplementary Figure 6. Quasiparticle dispersion for *x*=1.2 and 1.0. **a** and **b** ARPES data that were used to obtain QP dispersions of x = 1.2 and 1.0 data, respectively, in Fig. 2h.

Supplementary Note 6: Comparison of ARPES data and calculated spectral functions.

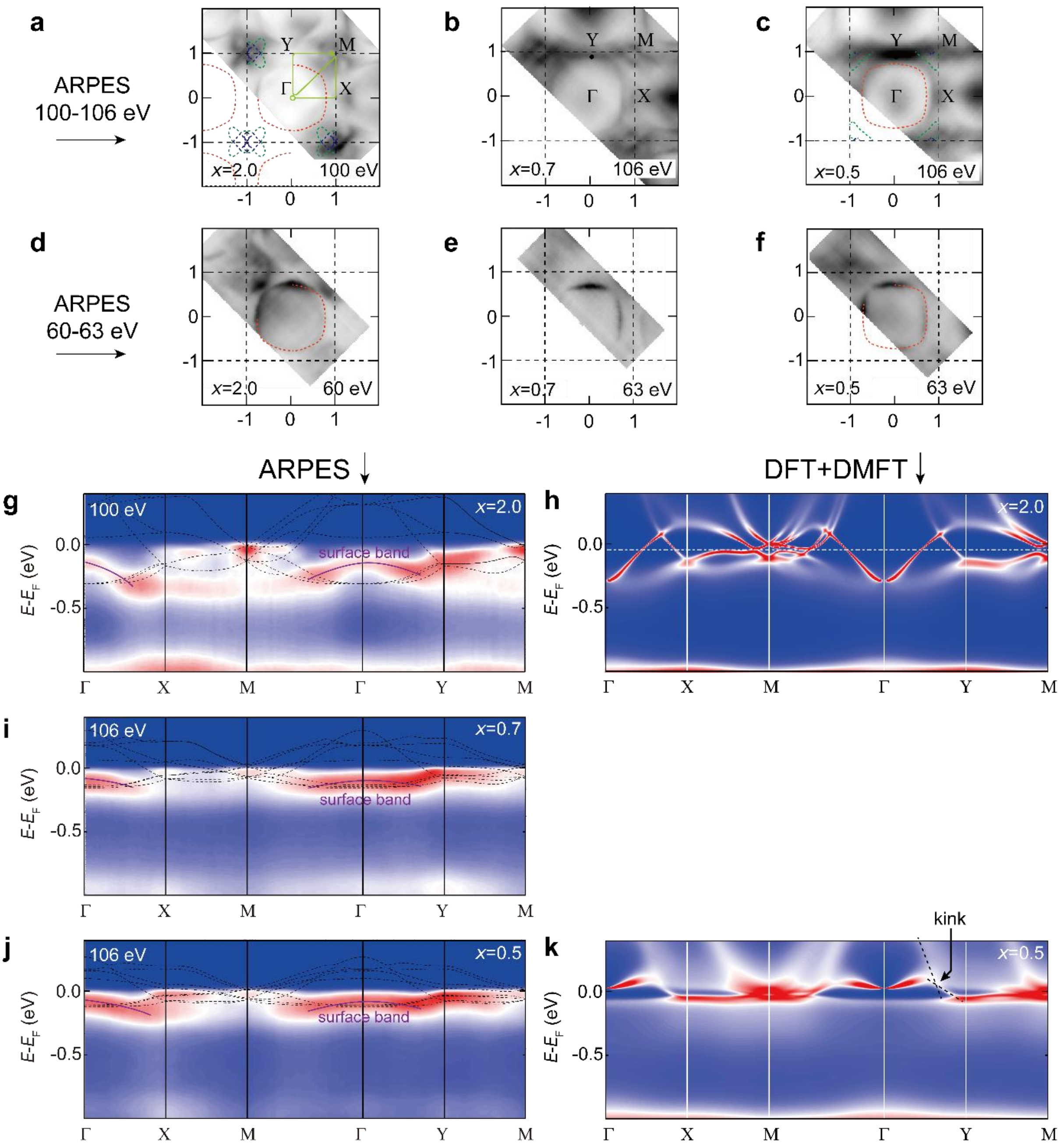

Supplementary Figure 7. Fermi surface (FS) maps and *k*-resolved spectral functions. **a-f** FS maps for *x*=2.0, 0.7, and 0.5 obtained with 100 (106) eV (**a-c**) and 60 (63) eV (**d-f**), respectively. The dashed lines indicate the calculated FS using DFT. **g-k** *k*-resolved spectral functions for *x*=2.0, 0.7, and 0.5 obtained from ARPES (**g, i,** and **j**) and DFT+DMFT calculations (**h, k**). The overlaid dashed lines in ARPES data indicate the renormalized DFT band structures.

We have 3 doping cases (x = 2.0, 0.7, 0.5) for the full spectral function in the form of Fig. 1c taken with photon energies in the 100-106 eV range. DFT+DMFT calculation was conducted at every x = 0.5

points, so that the only two cases (x = 2, 0.5) can be directly compared with the ARPES data as shown in Supplementary Fig. 7. For x = 0.7 case, we overlay the renormalized DFT band structure of x = 1.0 case, and the renormalized factor is obtained from the interpolation between x = 0.5 and 1.0 case.

Supplementary Figs. 7a-7f show Fermi surface (FS) maps obtained with 100-103 (a-c) and 60-63 (d-f) eV photon energies, respectively. As we discussed above, overall FS are well captured by 100-103 eV data while the α band is more clearly seen in the 60-63 eV data. The dotted lines represent the calculated FS. The rounded hexagon shape of α band FS at x = 2.0 and x = 0.5 case is well reproduced by the calculated FS.

Supplementary Figs. 7g-7k show the band structure obtained from ARPES measurement (left panels; g, i, and j) and the corresponding DFT+DMFT result (right panels; h, k). At x = 2.0 case (Supplementary Figs. 7g and h), as we discussed above, the complex δ bands around the M point and the α band near the Y point are well described by DFT+DMFT calculation. The band dispersion along the Y(X)-M direction also well agrees with each other. At x = 0.5 case (Supplementary Figs. 7j and k), DFT+DMFT calculation consistently describes the strongly renormalized band structure near the Fermi level. The detailed band structure cannot be clearly resolved since x = 0.5 case is close to the MIT. However, the measured and calculated band dispersions along the Y(X)-M direction agree well with each other.

The coherent part is confined within a low energy range near the Fermi level due to the Hund's coupling effect (See Supplementary Note 7 and the references in the main text). At x = 2.0 case, the DMFT spectrum becomes very incoherent beyond ~0.2 eV. This genuine coherent part near the Fermi level becomes much narrower in x = 0.5 case since the energy window where the self-energy follows the Fermi liquid behavior becomes narrower (kink moves toward to the lower energy side). Although the e_g band actually extends to about -0.5 eV, the spectral function between -0.5 and -0.1 eV becomes very incoherent due to the large ImΣ(w) which comes from the Hund's coupling effect (See Supplementary Note 7). This behavior is also well captured in the ARPES data. The genuine coherent regime becomes narrower as x decreases. Although the renormalized DFT band structure (dotted lines) are well agree with ARPES and DFT+DMFT result at this coherent regime near the Fermi level, but it deviates from the DFT+DMFT result above and below this coherent regime because the renormalization factor (the slope of ReΣ) changes at the kink (deviation from the Fermi liquid behavior). This deviation (kink) is more clearly seen above the Fermi level, especially for the x = 0.5 case (Supplementary Fig. 7k). As we mentioned in our main text, the kink becomes clear and moves towards the lower energy side as the system become closer the MIT (as S content increases).

In the x = 2.0 case ($NiSe_2$), the additional electron like pocket around the Γ point is observed in the DFT+DMFT result. This very coherent band has mainly Se p character. The interaction (bonding) between Se and Ni atoms is somewhat overestimated at the Γ point in the DFT+DMFT calculation so that the band moves below the Fermi level. Although this Se p band is not observed in the ARPES data, the Ni e_g bands, which are of our interest, are well described by DFT+DMFT calculation. Since we are interested in the energy scale of the system, we focused on describing the effective mass of Ni d orbitals and the metal-insulator transition observed in the experiment.

Supplementary Note 7: Momentum resolved spectral function with and without Hund's coupling.

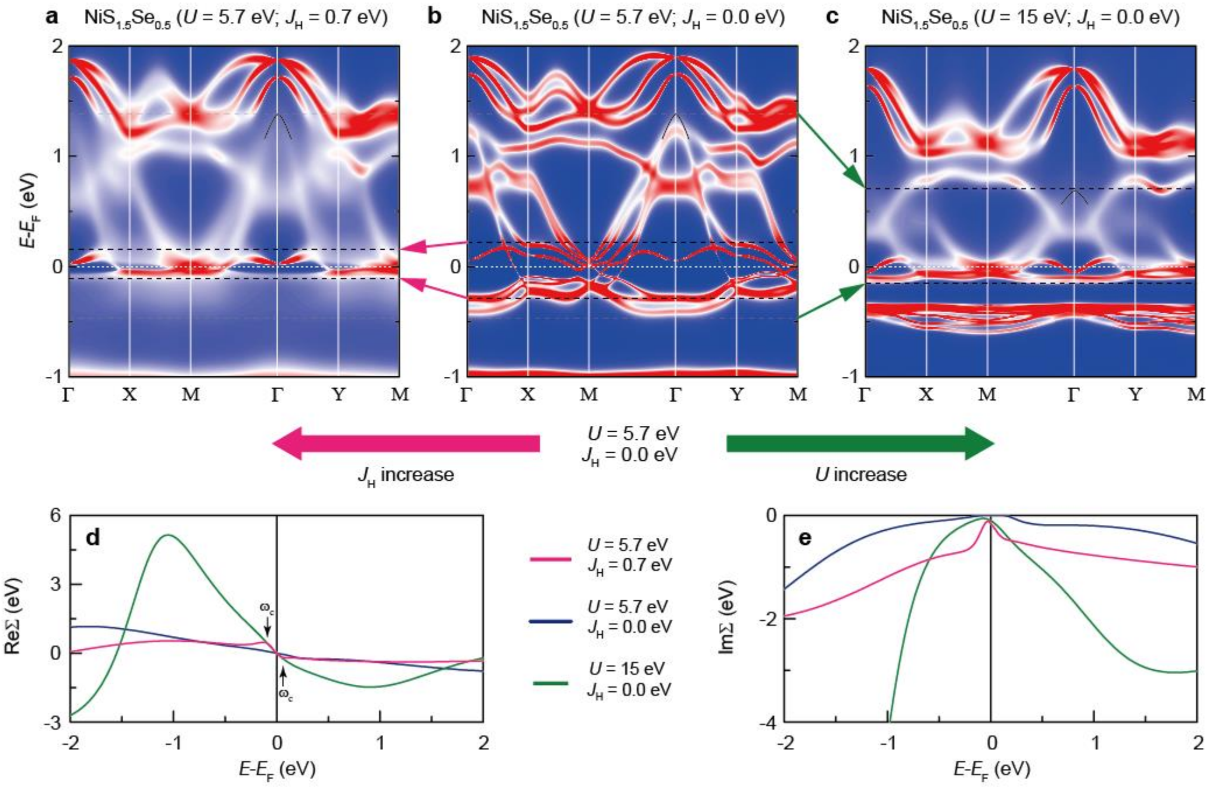

Supplementary Figure 8. Self-energy and the corresponding spectral function. DFT+DMFT spectral function of $NiS_{1.5}Se_{0.5}$ with **a** U=5.7 eV and J_H=0.7 eV, **b** U=5.7 eV and J_H=0 eV, and **c** U=15 eV and J_H=0 eV. **d** and **e** Real and imaginary parts of the self-energy for various cases, respectively.

As we discussed in the main text, J_H enhances the correlations differently from the U. When the Coulomb interaction U increases the correlation strength, the overall e_g band is renormalized as shown in Supplementary Fig. 8c. However, when the Hund's coupling J_H increases the correlation strength, only the small region near the Fermi level is strongly renormalized while the overall bandwidth of e_g orbitals remains almost the same, making kink features at ω_c. Although the e_g band actually extends to -0.5 eV in Supplementary Fig. 8a, the spectral function between -0.5 and -0.1 eV becomes very incoherent due to the large $\text{Im}\Sigma(\omega)$ (pink line) shown in Supplementary Fig 8e, and thus is not visible.

Supplementary Note 8: Kink position in the self-energy and spectral function

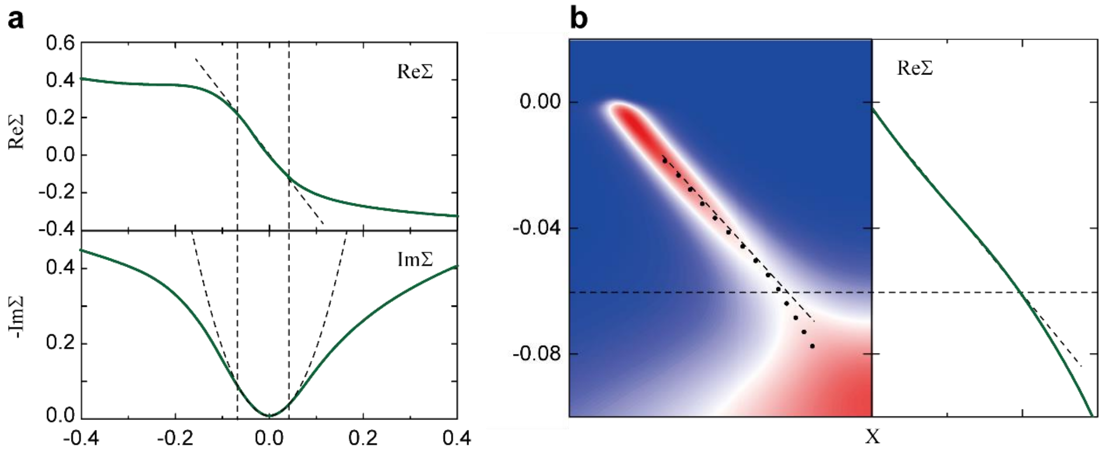

Supplementary Figure 9. Self-energy and the spectral function of NiS_1Se_1. a Self-energy obtained from DFT+DMFT calculation. The deviation from the Fermi liquid behavior in the self-energy makes kink feature in the spectral function. The horizontal dashed lines indicate the kink position defined with the real part of self-energy ($Re\Sigma(\omega)$). **b** A magnified view of α band and $Re\Sigma(\omega)$. Black dots indicates the quasiparticle band dispersion.

Although our DFT+DMFT calculation results consistently describe the experimental data over the phase diagram, including the *k*-integrated spectral function (Fig. 1d) and effective mass (Fig. 2k), it is not easy to define the kink position directly from the calculated spectral function. Due to the small energy scale of the kink, it is difficult to describe the exact position of the kink in the α band. In addition, the sharp feature of kink can be lost in the analytic continuation process. However, it is reasonable and possible to define the kink position from the self-energy ($\Sigma(\omega)$) behavior since the kink is an effect from $\Sigma(\omega)$. Therefore, we defined the kink position with the $Re\Sigma(\omega)$ at which the $Re\Sigma(\omega)$ starts to deviate from the linear behavior (the renormalization factor changes).

Supplementary Fig. 9 shows the calculated self-energy and spectral function of NiS_1Se_1. The horizontal dashed lines in Supplementary Fig. 9a indicate the kink position defined with the $Re\Sigma(\omega)$. Above this energy scale, the self-energy starts to deviate from the Fermi liquid behavior. The change of slope (renormalization factor) in $Re\Sigma(\omega)$ makes kink feature in the spectral function. The black dots in Supplementary Fig. 9b indicate the quasiparticle band dispersion. Above the kink energy scale defined with $Re\Sigma(\omega)$, the slope of quasiparticle band dispersion changes due to the different renormalization. In addition to the change in band dispersion, the spectral functions becomes very incoherent above the kink energy scale due to the large $Im\Sigma(\omega)$ originating from the Hund's coupling effect (See Supplementary Note 7).

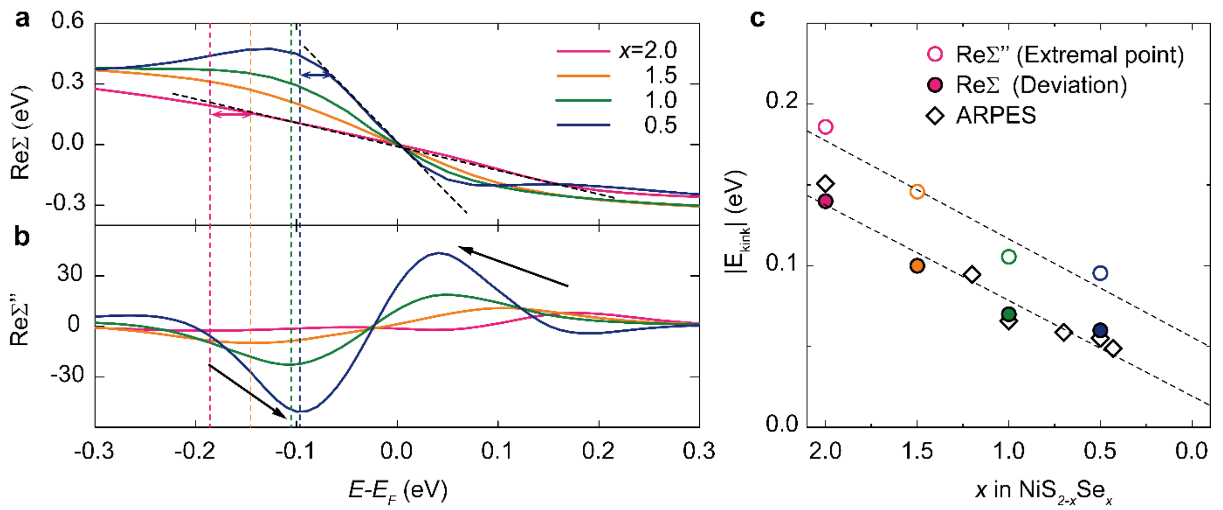

Supplementary Figure 10. Kink position defined with the second derivative of ReΣ (ReΣ'') a-b Real part of the self-energy ReΣ and its second derivative ReΣ''. The vertical lines indicate the kink position defined by using ReΣ''. **c** kink energy scale obtained from ARPES, ReΣ (deviation from the Fermi liquid behavior), and ReΣ'' (extremal point).

We also tried to define the kink position by using an extremal point of ReΣ'' (the second derivative of ReΣ), where the band dispersion changes most abruptly. The vertical dashed lines in Supplementary Figs. 10a and 10b indicate the kink defined by using ReΣ''. In this way, the kink energy scale obtained from DFT+DMFT calculation is slightly higher than that of ARPES (The colored horizontal bidirectional arrows indicate the difference between the kink defined by using the deviation from linear behavior and the extremal point of ReΣ''). However, they are just vertically shifted up from the kinks defined by using the deviation from the linear behavior of ReΣ (or the ARPES results) as shown in Supplementary Fig. 10c. Overall doping dependent evolution of the energy scale does not change (the slope remains almost the same). Regardless of how the kink position is defined, our DFT+DMFT calculation well describes the evolution of the energy scale as a function of the doping content *x*.

Supplementary Note 9: Optical conductivity of $NiS_{2-x}Se_x$

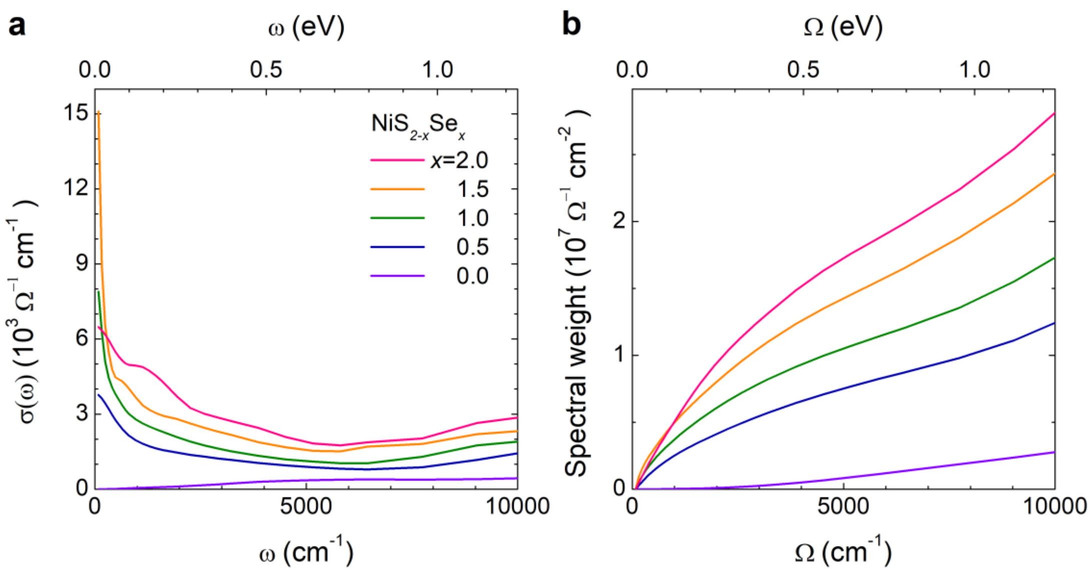

Supplementary Figure 11. Calculated optical conductivity from DFT+DMFT calculation. a Doping dependent optical conductivity **b** Spectral weight, the integrated optical conductivity up to Ω**.**

The doping dependent optical conductivity $\sigma(\omega)$ of $NiS_{2-x}Se_x$ shows a typical behavior of Mott systems. The Drude peak is gradually renormalized as the system approaches the MIT with S doping. The overall spectral weight also gets suppressed. Supplementary Fig. 11b shows the spectral weight which is the integrated optical conductivity, $\int_0^\Omega \sigma(\omega)d\omega$. The area under the Drude part of $\sigma(\omega)$ is proportional to the electron's kinetic energy. Supplementary Fig. 11 clearly shows that the overall energy scale is suppressed as the system approaches the MIT. This suppression allows us to observe the evolution of kink features in $NiS_{2-x}Se_x$ system. The high-resolution optical conductivity study would be interesting to further analyze the kink feature observed in ARPES data [6]

Supplementary Note 10: Crystal structure used in the calculation (cif format)

NiS_2 (*x* = 0.0)

```
_cell_length_a       5.689903
_cell_length_b       5.689903
_cell_length_c       5.689903
_cell_angle_alpha     90.000000
_cell_angle_beta       90.000000
_cell_angle_gamma     90.000000
_cell_measurement_temperature 0.0
_diffrn_ambient_temperature 0.0
_symmetry_space_group_name_H-M              'Pa-3     '
_symmetry_space_group_number   205

loop_
_symmetry_equiv_pos_as_xyz
     +x,+y,+z
     -x+1/2,-y,+z+1/2
     -x,+y+1/2,-z+1/2
     +x+1/2,-y+1/2,-z
     +z,+x,+y
     +z+1/2,-x+1/2,-y
     -z+1/2,-x,+y+1/2
     -z,+x+1/2,-y+1/2
     +y,+z,+x
     -y,+z+1/2,-x+1/2
     +y+1/2,-z+1/2,-x
     -y+1/2,-z,+x+1/2
     -x,-y,-z
     +x+1/2,+y,-z+1/2
     +x,-y+1/2,+z+1/2
     -x+1/2,+y+1/2,+z
     -z,-x,-y
     -z+1/2,+x+1/2,+y
     +z+1/2,+x,-y+1/2
     +z,-x+1/2,+y+1/2
     -y,-z,-x
     +y,-z+1/2,+x+1/2
     -y+1/2,+z+1/2,+x
     +y+1/2,+z,-x+1/2
loop_
_atom_site_label
_atom_site_type_symbol
_atom_site_fract_x
_atom_site_fract_y
_atom_site_fract_z
_atom_site_U_iso_or_equiv
Ni001    Ni    0.00000000    0.00000000    0.00000000    0.05000000
S0002    S     0.39500000    0.39500000    0.39500000    0.05000000
```

$NiS_{1.5}Se_{0.5}$ (*x* = 0.5)

```
_cell_length_a       5.754927
_cell_length_b       5.754927
_cell_length_c       5.754927
_cell_angle_alpha     90.000001
_cell_angle_beta       90.000001
_cell_angle_gamma     90.000001
```

```
_cell_measurement_temperature 0.0
_diffrn_ambient_temperature 0.0
_symmetry_space_group_name_H-M              'R-3       '
_symmetry_space_group_number   148
_space_group_crystal_system rhombohedral

loop_
_symmetry_equiv_pos_as_xyz
    +x,+y,+z
    +z,+x,+y
    +y,+z,+x
    -x,-y,-z
    -z,-x,-y
    -y,-z,-x
loop_
_atom_site_label
_atom_site_type_symbol
_atom_site_fract_x
_atom_site_fract_y
_atom_site_fract_z
_atom_site_U_iso_or_equiv
Ni001   Ni   0.00000000   0.00000000   0.00000000   0.05000000
Ni002   Ni   0.00000000   0.50000000   0.50000000   0.05000000
S0003   S    0.10466373   0.89277307   0.39336381   0.05000000
Se004   Se   0.37903657   0.37903657   0.37903657   0.05000000
```

NiS_1Se_1 (x = 1.0)

```
_cell_length_a       5.819952
_cell_length_b       5.819952
_cell_length_c       5.819952
_cell_angle_alpha     90.000000
_cell_angle_beta      90.000000
_cell_angle_gamma     90.000000
_cell_measurement_temperature 0.0
_diffrn_ambient_temperature 0.0
_symmetry_space_group_name_H-M              'P21/c    '
_symmetry_space_group_number     14

loop_
_symmetry_equiv_pos_as_xyz
    +x,+y,+z
    -x+1/2,-y,+z+1/2
    -x,-y,-z
    +x+1/2,+y,-z+1/2
loop_
_atom_site_label
_atom_site_type_symbol
_atom_site_fract_x
_atom_site_fract_y
_atom_site_fract_z
_atom_site_U_iso_or_equiv
Ni001   Ni   0.50000000   0.00000000   0.50000000   0.05000000
Ni002   Ni   0.50000000   0.50000000   0.00000000   0.05000000
S0003   S    0.39448455   0.10494396   0.89233821   0.05000000
Se004   Se   0.12128780   0.62023957   0.88010074   0.05000000
```

$NiS_{0.5}Se_{1.5}$ (*x* = 1.5)

```
_cell_length_a       5.884976
_cell_length_b       5.884976
_cell_length_c       5.884976
_cell_angle_alpha     90.000000
_cell_angle_beta      90.000000
_cell_angle_gamma     90.000000
_cell_measurement_temperature 0.0
_diffrn_ambient_temperature 0.0
_symmetry_space_group_name_H-M              'R-3       '
_symmetry_space_group_number   148
_space_group_crystal_system rhombohedral

loop_
_symmetry_equiv_pos_as_xyz
     +x,+y,+z
     +z,+x,+y
     +y,+z,+x
     -x,-y,-z
     -z,-x,-y
     -y,-z,-x
loop_
_atom_site_label
_atom_site_type_symbol
_atom_site_fract_x
_atom_site_fract_y
_atom_site_fract_z
_atom_site_U_iso_or_equiv
Ni001   Ni   0.00000000   0.00000000   0.00000000   0.05000000
Ni002   Ni   0.00000000   0.50000000   0.50000000   0.05000000
Se003   Se   0.12194252   0.87937351   0.37957938   0.05000000
S0004   S    0.39382867   0.39382867   0.39382867   0.05000000
```

$NiSe_2$ (*x* = 2.0)

```
_cell_length_a       5.960398
_cell_length_b       5.960398
_cell_length_c       5.960398
_cell_angle_alpha     90.000000
_cell_angle_beta      90.000000
_cell_angle_gamma     90.000000
_cell_measurement_temperature 0.0
_diffrn_ambient_temperature 0.0
_symmetry_space_group_name_H-M              'Pa-3      '
_symmetry_space_group_number   205

loop_
_symmetry_equiv_pos_as_xyz
     +x,+y,+z
     -x,-y,-z
     -z,-x,-y
     -y,-z,-x
     +y,+z,+x
     +z,+x,+y
     +z+1/2,-x+1/2,-y
     -z+1/2,-x,+y+1/2
     +z,-x+1/2,+y+1/2
     -x,+y+1/2,-z+1/2
```

-y,+z+1/2,-x+1/2
+y+1/2,-z+1/2,-x
+y+1/2,+z,-x+1/2
-y+1/2,-z,+x+1/2
-y+1/2,+z+1/2,+x
+y,-z+1/2,+x+1/2
-x+1/2,+y+1/2,+z
-z,+x+1/2,-y+1/2
+z+1/2,+x,-y+1/2
-z+1/2,+x+1/2,+y
-x+1/2,-y,+z+1/2
+x+1/2,-y+1/2,-z
+x,-y+1/2,+z+1/2
+x+1/2,+y,-z+1/2
loop_
_atom_site_label
_atom_site_type_symbol
_atom_site_fract_x
_atom_site_fract_y
_atom_site_fract_z
_atom_site_U_iso_or_equiv
Ni001 Ni 0.00000000 0.00000000 0.00000000 0.05000000
Se002 Se 0.38400000 0.38400000 0.38400000 0.05000000

References

[1] Bouchard, R. J., The preparation of single crystals of FeS_2, CoS_2, and NiS_2 pyrites by chlorine transport. *J. Cryst. Growth* **2**, 40 (1968).

[2] Matsuura, M. *et al.*, Magnetic Phase Diagram and Metal-Insulator Transition of $NiS_{2-x}Se_x$. *J. Phys. Soc. Jpn.* **69**, 1503 (2000).

[3] Matsuura, A. Y. *et al.*, Electronic structure and the metal-insulator transition in $NiS_{2-x}Se_x$. *Phys. Rev. B* **53**, R7584 (1996).

[4] Matsuura, A. Y. *et al.*, Metal-insulator transition in $NiS_{2-x}Se_x$ and the local impurity self-consistent approximation model. *Phys. Rev. B* **58**, 3690 (1998).

[5] Xu, H. C. *et al.*, Direct Observation of the Bandwidth Control Mott Transition in the $NiS_{2-x}Se_x$ Multiband System. *Phys. Rev. Lett.* **112**, 087603 (2014).

[6] Dang, H. T. *et al.*, Band Structure and Terahertz Optical Conductivity of Transition Metal Oxides: Theory and Application to $CaRuO_3$. *Phys. Rev. Lett.* **115**, 107003 (2015).

[7] Borghi, G. et al., Surface Dead Layer for Quasiparticles Near a Mott Transition. *Phys. Rev. Lett.* **102**, 066806 (2009).

[8] Iwaya, K. et al., Evolution of local electronic states from a metal to a correlated insulator in a $NiS_{2-x}Se_x$ solid solution *Phys. Rev. B* **70**, 161103 (2004).

[9] Levy, G. et al., Deconstruction of resolution effects in angle-resolved photoemission. *Phys. Rev. B* **90**, 045150 (2014).
